# Associations of workload, work satisfaction, change fatigue, and presenteeism with quiet quitting among healthcare professionals: a structural equation modeling study

**DOI:** 10.3389/fpubh.2026.1936291

**Published:** 2026-10-21

**Authors:** Birol Yetim, Seval Selvi Sarıgül, İsmail Biçer, Yusuf Çelik

**Affiliations:** 1 Department of Health Care Management, Faculty of Health Sciences, Mus Alparslan University, Muş, Türkiye; 2 Department of Health Care Management, Faculty of Economics and Administrative Sciences, Erzincan Binali Yıldırım University, Erzincan, Türkiye; 3 Çivril Atasay Kamer Vocational School, Health Institutions Management, Pamukkale University, Denizli, Türkiye; 4 Department of Healthcare Management, Faculty of Health Sciences, Acıbadem Mehmet Ali Aydınlar University, İstanbul, Türkiye; 5 Department of Healthcare Management, Graduate School of Health Sciences, Acıbadem Mehmet Ali Aydınlar University, İstanbul, Türkiye

**Keywords:** change fatigue, healthcare professionals, presenteeism, quiet quitting, structural equation model, work satisfaction, workload

## Abstract

**Background:**

Quiet quitting is an increasingly relevant concern in healthcare settings, where high workload, repeated organizational change, and presenteeism may be associated with reduced motivation, engagement, and service quality. This study examined the direct and indirect associations of workload, work satisfaction, change fatigue, and presenteeism with quiet quitting among healthcare professionals.

**Methods:**

A cross-sectional study was conducted between July and September 2025 among healthcare professionals employed for at least 1 year at a single public hospital in Denizli, Türkiye. The sample included 329 participants: 146 physicians, 117 nurses, and 66 other healthcare professionals. Data were collected using a personal information form and validated scales measuring workload, work satisfaction, change fatigue, presenteeism, and quiet quitting. Descriptive statistics, correlation analysis, confirmatory factor analysis, structural equation modeling, and mediation analysis were performed.

**Results:**

The structural model showed mixed fit and explained 55.6% of the variance in quiet quitting. Workload showed the strongest association with quiet quitting (β = 0.589, *p* < 0.001), and work satisfaction was negatively associated with quiet quitting (β = −0.097, *p* = 0.001). The direct association between presenteeism and quiet quitting was positive but marginal and did not meet strict significance criteria, as the bootstrap confidence interval included zero (B = 0.353, 95% CI [−0.008, 0.680]; β = 0.233, *p* =  0.039) and was not robust in sensitivity analyses. Change fatigue was positively associated with workload (β = 0.185, *p* = 0.011) and presenteeism (β = 0.539, *p* < 0.001), but was not directly associated with quiet quitting. Change fatigue showed significant indirect associations with quiet quitting through workload, through presenteeism, and through the sequential pathway of workload and work satisfaction (total effect β = 0.383, *p* < 0.001).

**Conclusion:**

Workload was positively associated with quiet quitting, whereas greater work satisfaction was associated with lower quiet quitting. The association between presenteeism and quiet quitting was positive but marginal and requires replication before firm conclusions can be drawn. Workload was the association that remained stable across all model specifications and professional groups, suggesting that manageable workload distribution and organizational support during periods of change are the most promising targets for further study. Given the cross-sectional design, the temporal direction of these relationships cannot be determined. Longitudinal and multicenter studies are needed to clarify the observed relationships.

## 1 Introduction

The healthcare sector directly impacts human life and places significant physical and psychological burdens on healthcare workers due to its high workload and responsibility (1, 2). Recent technological advancements, changes in organizational structures, personnel shortages, and rising patient demands have further increased workload and stress levels among healthcare workers (3). This environment is associated not only with burnout and reduced productivity but also with quiet quitting, defined as “a process in which employees, despite their physical presence in the work environment, lose their organizational commitment and motivation” (4–6). Quiet quitting involves avoiding cognitive and emotional investment beyond minimum job responsibilities and is associated with decreased productivity and a weakened sense of belonging within the organization (7). It is considered a critical contemporary issue due to its association with reduced organizational commitment and psychological withdrawal behaviors (8).

Quiet quitting should be distinguished from several related occupational concepts. Unlike turnover intention, which refers to an employee’s conscious willingness or plan to leave an organization, quiet quitting occurs while the employee remains in the job and limits effort to formally required duties (8). Burnout represents a psychological syndrome characterized by exhaustion, cynicism, and reduced professional efficacy (9), whereas work disengagement refers more broadly to the withdrawal of cognitive, emotional, and physical investment from work (10). Organizational silence, in contrast, involves intentionally withholding work-related opinions, concerns, or information (11). Quiet quitting may overlap with these experiences and with other withdrawal behaviors, such as absenteeism or reduced effort, but it does not necessarily imply an intention to leave the organization (8).

Nevertheless, quiet quitting remains a conceptually debated and still evolving construct. Although it is frequently interpreted as disengagement or a negative employee attitude, it may also represent a coping response through which employees establish professional boundaries, limit unpaid or excessive work demands, and protect their work–life balance (12). Accordingly, performing formally assigned duties without undertaking additional responsibilities should not automatically be interpreted as withdrawal or diminished commitment. The construct has been described as a combination of reduced effort, disengagement, dissociation, and boundary-setting; however, its distinctiveness from these established concepts remains open to question (10). Therefore, the meaning of quiet quitting should be interpreted in relation to employees’ working conditions and the organizational context.

Quiet quitting is particularly significant in healthcare because of its potential implications for service quality, patient safety, and institutional performance (13). Healthcare workers often experience stress, high workload, and uncertainty, which can lead to negative emotions such as loss of motivation (14), unwillingness to work, and burnout (15, 16). These negative situations may lead employees to perform their duties as mere formal obligations (17). Unlike traditional resignation, quiet quitting manifests internally, progresses insidiously, and can have long-term organizational consequences. Identifying the organizational and psychological factors associated with quiet quitting is therefore essential to maintaining employee commitment and sustaining healthcare services (18).

Psychosocial and structural factors shape the emergence of quiet quitting among healthcare workers. Change fatigue, defined as emotional exhaustion resulting from frequent and intense organizational changes (19), reduces employees’ commitment, particularly in high-stress healthcare environments (20, 21). Healthcare workers under constant change pressure tend to limit their effort to formal obligations, avoiding extra work and organizational identification, which reinforces quiet quitting through psychological withdrawal behaviors (22). Simultaneously, excessive workload from structural issues such as increasing patient numbers, inadequate staffing, and long working hours exhausts physical and mental resources, leading to burnout and job alienation (23). Increased workload weakens emotional commitment (24, 25) and professional motivation (26–28), contributing to quiet quitting (6, 23, 29). Presenteeism, defined as attending work physically while being psychologically detached (30, 31), reduces short-term organizational performance (32), and long-term work satisfaction (33, 34) and organizational commitment (35–37). Empirical studies indicate that presenteeism increases emotional exhaustion and accelerates psychological withdrawal, thereby facilitating quiet quitting (5). Conversely, work satisfaction acts as a protective factor; employees with higher satisfaction maintain organizational commitment and resist quiet quitting despite increasing workload and change pressures (23, 38, 39).

Recent evidence suggests that quiet quitting has become an increasingly important issue in healthcare organizations, particularly in hospital settings with high workloads and emotionally demanding working conditions. Although empirical studies on quiet quitting among healthcare professionals remain limited, available evidence indicates that quiet quitting may adversely affect patient care quality, organizational commitment, healthcare service effectiveness, patient satisfaction, and workforce sustainability. Furthermore, recent evidence has shown that burnout is positively associated with quiet quitting, whereas higher job satisfaction reduces employees’ tendency toward quiet quitting. Therefore, identifying the organizational and psychosocial determinants of quiet quitting has become an important priority for healthcare management research and practice (6, 40).

### 1.1 Theoretical framework and hypotheses

The proposed model is grounded primarily in Job Demands–Resources (JD–R) theory and is further supported by Conservation of Resources (COR) theory. JD–R theory proposes that job characteristics can be broadly classified as job demands and job resources. Job demands require sustained physical or psychological effort and may be accompanied by physiological or psychological costs, whereas job resources facilitate the achievement of work goals and support employee wellbeing and motivation (41). Within the present model, workload represents a central job demand, while change fatigue reflects the strain associated with repeated organizational changes. The accumulation of these demands may lead to energy depletion, presenteeism, and psychological withdrawal. Work satisfaction, although not a job resource itself, reflects employees’ positive evaluation of their work and may decline when job demands become excessive.

COR theory complements this perspective by proposing that individuals seek to obtain, maintain, and protect valued resources, including time, energy, emotional capacity, and psychological wellbeing. Resource loss is particularly salient when employees face persistent demands without adequate opportunities for recovery or resource replenishment (42). From this perspective, change fatigue and workload may threaten or deplete employees’ resources, while presenteeism and restricting work effort to formally required duties may emerge as attempts to conserve remaining resources. Reduced work satisfaction may similarly accompany this resource-depletion process and be related to greater psychological withdrawal. Together, JD–R and COR theories provide a framework for examining the direct and indirect relationships among change fatigue, workload, presenteeism, work satisfaction, and quiet quitting.

Against this theoretical background, the present study examines the direct and indirect associations of workload, work satisfaction, change fatigue, and presenteeism with quiet quitting among healthcare workers using structural equation modeling. Although previous studies have generally examined these variables, limited research has investigated their complex, simultaneous interrelationships. This study addresses this gap by examining quiet quitting as a multidimensional process and investigating the relevant psychosocial mechanisms within the healthcare context. Based on this theoretical framework and the relationships included in the proposed model, the following hypotheses were formulated:

*H1a:* Change fatigue is positively associated with presenteeism.

*H1b:* Change fatigue is positively associated with workload.

*H1c:* Change fatigue is positively associated with quiet quitting.

*H2:* Workload is positively associated with quiet quitting.

*H3:* Presenteeism is positively associated with quiet quitting.

*H4*: Workload is negatively associated with work satisfaction.

*H5:* Work satisfaction is negatively associated with quiet quitting.

*H6a:* Change fatigue is indirectly associated with quiet quitting through presenteeism.

*H6b:* Change fatigue is indirectly associated with quiet quitting through workload.

*H6c:* Change fatigue is indirectly associated with quiet quitting through the sequential pathway of workload and work satisfaction.

*H7:* Workload is indirectly associated with quiet quitting through work satisfaction.

## 2 Methods

### 2.1 Objectives

This study aimed to examine the direct and indirect associations of workload, work satisfaction, change fatigue, and presenteeism with quiet quitting among healthcare professionals using SEM. Specifically, the study examined the direct associations, in the proposed model; the indirect associations of change fatigue with quiet quitting through presenteeism and workload, including the sequential pathway through workload and work satisfaction; and the indirect association of workload with quiet quitting through work satisfaction.

### 2.2 Population and sample

This observational, cross-sectional, single-center study was conducted between 1 July and 30 September 2025, among healthcare professionals employed at a public hospital in Denizli, Türkiye. The hospital employed 2,250 healthcare professionals, including 999 physicians, 797 nurses, and 454 other healthcare professionals. Proportionate quota sampling was used based on these three occupational groups. The quota for each group was determined proportionally to its representation in the hospital workforce. Participant selection within each group was not random; eligible healthcare professionals who voluntarily agreed to participate were recruited until the corresponding quota was reached. Healthcare professionals employed at the hospital for at least 1 year who voluntarily agreed to participate were eligible for inclusion. Those with less than 1 year of employment and those who returned incomplete questionnaires were excluded.

Questionnaires were distributed to 383 eligible healthcare professionals, of whom 329 participated, yielding a response rate of 85.9%. The final sample comprised 146 physicians, 117 nurses, and 66 other healthcare professionals. All 329 completed questionnaires were included in the analysis. Written informed consent was obtained from all participants before data collection.

Sample adequacy was evaluated in relation to model complexity and statistical power. The final path model contained 11 freely estimated parameters, yielding approximately 29.9 observations per parameter at *N* = 329. A fully latent model incorporating all 35 indicators would require 93 freely estimated parameters, corresponding to approximately 3.54 observations per parameter (80). Model complexity relative to the available sample size therefore informed the decision to estimate the structural relationships using composite scores, while evaluating measurement properties separately through confirmatory factor analysis (Table 1). Statistical power was evaluated using a Monte Carlo sensitivity analysis following Muthén and Muthén (43). Using the model-implied covariance matrix as the population matrix, 1,000 datasets of *N* = 329 were generated, and the model was re-estimated in each replication. Empirical power at α = 0.05 was 0.999 for the indirect association of change fatigue with quiet quitting through presenteeism, 0.938 for the indirect association through workload, and 1.000 for the total indirect association. Power was lower for the indirect association of workload with quiet quitting through work satisfaction (0.636) and the sequential indirect association of change fatigue with quiet quitting through workload and work satisfaction (0.326). These findings support sample adequacy for detecting the principal indirect associations under the simulation assumptions, while indicating limited power for the two smallest indirect associations.

**Table 1:** Measurement model: standardized loadings, reliability, convergent, and discriminant validity (*N* = 329).

Panel A. Standardized factor loadings, composite reliability, and average variance extracted			
Factor and items	λ	CR	AVE
Workload (11 items)	0.608–0.852	0.942	0.601
Work satisfaction (5 items)	0.410–0.798	0.738	0.375
Quiet quitting (7 items)	0.761–0.860	0.932	0.663
Change fatigue (6 items)	0.765–0.915	0.926	0.676
Presenteeism (6 items)	0.551–0.757	0.841	0.470

Panel B. Discriminant validity					
Construct	WL	WS	QQ	CF	PRE
Workload (WL)	**0.775**	0.225	0.695	0.178	0.220
Work satisfaction (WS)	−0.321	**0.613**	0.223	0.137	0.210
Quiet quitting (QQ)	0.719	−0.404	**0.814**	0.376	0.431
Change fatigue (CF)	0.191	−0.079	0.388	**0.822**	0.603
Presenteeism (PRE)	0.233	−0.145	0.491	0.612	**0.685**

λ = standardized factor loading; CR = composite reliability; AVE = average variance extracted. In Panel B, diagonal values (in bold) are the square root of AVE, values below the diagonal are latent correlations, and values above the diagonal are heterotrait-monotrait ratios. Model fit: scaled χ^2^(541) = 1007.87, *p* < 0.001; CFI = 0.914; TLI = 0.906; RMSEA = 0.061, 90% CI [0.055, 0.067]; SRMR = 0.075.

One of the researchers distributed the questionnaires in person, and staff completed them in staff rest rooms at times that did not interfere with their duties. The researchers collected no names, personnel numbers, or other identifying information. Each questionnaire was assigned a numerical code solely for data entry and analysis, and participants returned completed questionnaires directly to the researcher. These procedures preserved anonymity and reduced concerns about identification when responding to sensitive organizational questions. The manuscript was reviewed and revised with consideration of the Strengthening the Reporting of Observational Studies in Epidemiology (STROBE) recommendations (44).

### 2.3 Analysis

Descriptive statistics, including frequencies, percentages, means, and standard deviations, were calculated for sociodemographic and occupational characteristics and study variables. Missing data were assessed at both the item and participant levels. Univariate distributions were examined using skewness and kurtosis coefficients, and multivariate normality was assessed using Mardia’s multivariate skewness and kurtosis tests. Potential univariate outliers were screened using an absolute z-score threshold of 3.29. Potential multivariate outliers were identified using Mahalanobis distance with a chi-square criterion of *p* < 0.001 and five degrees of freedom. Flagged observations were examined for possible data-entry errors and plausible response patterns rather than automatically excluded. A sensitivity analysis excluding multivariate outliers was conducted to assess the robustness of the structural relationships. Multicollinearity was evaluated using variance inflation factors, tolerance statistics, and the condition number of the predictor correlation matrix.

The previously established factor structures of the instruments were evaluated using confirmatory factor analysis (CFA). Internal consistency was assessed using Cronbach’s alpha and McDonald’s omega. Composite reliability and average variance extracted were calculated, and discriminant validity was evaluated using the Fornell–Larcker criterion and heterotrait-monotrait ratios (79). Potential common method variance was examined using Harman’s single-factor test and by comparing a single-factor CFA model with the hypothesized five-factor measurement model.

Pearson’s correlation analysis examined bivariate associations among the study variables. Direct and indirect associations were examined using a simultaneous path model based on composite scores. CFA and path models were estimated using robust maximum likelihood (MLR) to address departures from multivariate normality. Model fit was evaluated using the scaled chi-square statistic, CFI, TLI, RMSEA, and SRMR. CFI and TLI values of 0.90 or higher and RMSEA and SRMR values of 0.08 or lower were used as guidelines for acceptable fit (81).

Indirect associations were evaluated using non-parametric bootstrapping with 5,000 resamples, and bias-corrected 95% confidence intervals were calculated. An indirect association was considered statistically significant when its confidence interval did not include zero. Supplementary analyses examined the robustness of the findings after adjustment for occupational and sociodemographic characteristics. Multigroup path analysis was also conducted to examine differences across professional groups by comparing models with freely estimated and equality-constrained structural paths.

All analyses were conducted in R version 4.6.1 (77) using RStudio. The principal packages were lavaan version 0.7–2 (78) for CFA and path modeling, psych version 2.6.5 for descriptive and psychometric analyses, and semTools version 0.5–9 for supplementary measurement analyses. Statistical significance was evaluated at a two-sided α level of 0.05.

### 2.4 Data collection tools

Data were collected using a Personal Information Form and five scales: the Workload Scale, Work Satisfaction Scale, Change Fatigue Scale, Stanford Presenteeism Scale (SPS 6), and Quiet Quitting Scale.

*Personal Information Form:* Comprised 11 items covering age, gender, occupation, marital status, education, department, total employment duration, weekly working hours, number of shifts, contract type, and total employment time.

*Workload Scale:* Developed by Peterson et al. (45) and validated in Turkish by Derya (46), this unidimensional 11-item, 5-point Likert scale measures workload intensity. Items are rated from 1 (“strongly disagree”) to 5 (“strongly agree”). Item 7 is reverse-scored. Item scores were averaged to obtain an overall score ranging from 1 to 5. Higher scores indicate greater workload.

*Work Satisfaction Scale:* Originally developed by Brayfield and Rothe (47) and shortened to five items by Judge et al. (48), this unidimensional five-item scale was validated in Turkish by Keser and Bilir (49). Items are rated on a 5-point Likert scale ranging from 1 (“strongly disagree”) to 5 (“strongly agree”). Items 3 and 5 are reverse-scored. Item scores were averaged to obtain an overall score ranging from 1 to 5. Higher scores indicate greater work satisfaction.

*Change Fatigue Scale:* Developed by Bernerth et al. (19) and validated in Turkish by Sarıgül and Uğurluoğlu (20), this six-item, unidimensional, 5-point Likert scale measures the cognitive and emotional impact of organizational changes. Items are rated from 1 (“strongly disagree”) to 5 (“strongly agree”). The scale contains no reverse-scored items. Item scores were averaged to obtain an overall score ranging from 1 to 5. Higher scores indicate greater change fatigue.

*Stanford Presenteeism Scale (SPS 6):* Developed by Koopman et al. (50) and validated in Turkish by Moç (51), this six-item, unidimensional 5-point Likert scale measures employees’ inability to be fully present at work. Items are rated from 1 (“strongly disagree”) to 5 (“strongly agree”). No items were reverse-scored in the version used in this study. Item scores were averaged to obtain an overall score ranging from 1 to 5. Higher scores indicate greater presenteeism.

*Quiet Quitting Scale:* The Quiet Quitting Scale, developed by Anand et al. (52) and adapted and validated in Turkish by Yıldız et al. (53), is a seven-item, unidimensional scale that measures the level of quiet quitting. Items are rated on a 5-point Likert scale ranging from 1 (“strongly disagree”) to 5 (“strongly agree”). The scale contains no reverse-scored items. Item scores were averaged to obtain an overall score ranging from 1 to 5. Higher scores indicate greater levels of quiet quitting.

Confirmatory factor analyses were conducted to assess the structural validity of all scales. Model fit was evaluated using index-specific criteria rather than a single cutoff value. CFI and TLI values of 0.90 or higher and RMSEA and SRMR values of 0.08 or lower were considered indicative of acceptable model fit. Internal consistency coefficients were 0.931 for quiet quitting, 0.942 for workload, 0.776 for work satisfaction, 0.840 for presenteeism, and 0.927 for change fatigue, indicating acceptable to excellent internal consistency.

## 3 Results

As shown in Table 2, the participants had a mean age of 33.21 ± 6.718 years (range: 22–56). Of the 329 healthcare professionals included in the study, 201 (61.1%) were female, and 128 (38.9%) were male. In terms of marital status, 175 participants (53.2%) were married, and 154 (46.8%) were single. Regarding educational background, 20 participants (6.1%) were high school graduates, 237 (72.0%) held an associate or undergraduate degree, 24 (7.3%) had a master’s degree, 7 (2.1%) held a doctorate, and 41 (12.5%) held a medical specialization. By profession, 146 participants (44.4%) were physicians, 117 (35.6%) were nurses, and 66 (20.1%) were other healthcare professionals. In terms of the departments where they worked, 94 participants (28.6%) worked in internal medicine units, 117 (35.6%) in surgical units, 13 (4.0%) in intensive care units, 43 (13.1%) in outpatient clinics, 27 (8.2%) in emergency departments, 8 (2.4%) in operating rooms, and 27 (8.2%) in other units. Regarding employment status, most participants (n = 311, 94.5%) were permanent staff, while 18 (5.5%) were contracted staff. Participants’ weekly working hours averaged 43.74 ± 2.010 h (range: 40–48), weekly on-call duties averaged 0.69 ± 0.630 (range: 0–3), and total professional experience in the healthcare sector averaged 8.46 ± 5.881 years (range: 1–30).

**Table 2:** Sociodemographic and occupational characteristics of the participants (*N* = 329).

Characteristic	*n*	%
Age, years (mean ± SD)	33.21 ± 6.718 (range: 22–56)	
Gender		
Female	201	61.1
Male	128	38.9
Marital status		
Married	175	53.2
Single	154	46.8
Educational status		
High school	20	6.1
Associate/Bachelor’s degree	237	72.0
Master’s degree	24	7.3
Doctoral degree	7	2.1
Specialization	41	12.5
Profession		
Physician	146	44.4
Nurse	117	35.6
Other	66	20.1
Department		
Internal medicine units	94	28.6
Surgical units	117	35.6
Intensive care unit	13	4.0
Outpatient clinic	43	13.1
Emergency department	27	8.2
Operating room	8	2.4
Other	27	8.2
Type of employment contract		
Permanent staff	311	94.5
Contracted staff	18	5.5
Weekly working hours (mean ± SD)	43.74 ± 2.010 (range: 40–48)	
Weekly number of on-call duties (mean ± SD)	0.69 ± 0.630 (range: 0–3)	
Professional experience in healthcare, years (mean ± SD)	8.46 ± 5.881 (range: 1–30)	

SD, Standard deviation.

Descriptive statistics, internal consistency coefficients, and bivariate correlations for the study variables are presented in Table 3. Among the study variables, quiet quitting had the highest mean score (mean = 4.41 ± 0.64), close to the upper end of the possible range of 1 to 5, with a median of 4.57 and a pronounced negative skew (−2.14). Change fatigue had the lowest mean score (mean = 2.87 ± 0.37), positioned near the scale midpoint, and also showed a marked negative skew (−2.14). Workload (mean = 3.51 ± 0.51) and presenteeism (mean = 3.25 ± 0.49) were positioned above the scale midpoint, and work satisfaction had a mean of 2.89 ± 0.62, near the midpoint. As no validated cutoff points are available for these instruments, scores are reported descriptively relative to the possible scale range rather than classified into categorical levels. Internal consistency was acceptable to excellent for all scales, with Cronbach’s alpha ranging from 0.776 to 0.942 and McDonald’s omega from 0.779 to 0.944. Workload showed a strong positive correlation with quiet quitting (r = 0.679), and presenteeism (r = 0.423) and change fatigue (r = 0.370) were also positively correlated with quiet quitting. Work satisfaction was negatively correlated with both workload (r = −0.242) and quiet quitting (r = −0.223), whereas its correlations with change fatigue (r = 0.047) and presenteeism (r = 0.028) were negligible (Table 3).

**Table 3:** Descriptive statistics, internal consistency, and correlations among the study variables (*N* = 329).

Variable	Possible range	Mean ± SD	Median	Observed range	Skew	Kurtosis	α	ω	1	2	3	4	5
1. Workload	1–5	3.51 ± 0.51	3.64	2.00–4.64	−0.33	−0.68	0.942	0.944	—				
2. Work satisfaction	1–5	2.89 ± 0.62	2.80	1.00–4.80	0.06	−0.10	0.776	0.779	−0.242	—			
3. Quiet quitting	1–5	4.41 ± 0.64	4.57	1.00–5.00	−2.14	6.23	0.931	0.932	0.679	−0.223	—		
4. Change fatigue	1–5	2.87 ± 0.37	3.00	1.00–4.33	−2.14	8.93	0.927	0.928	0.185	0.047	0.370	—	
5. Presenteeism	1–5	3.25 ± 0.49	3.33	1.00–4.67	−2.05	5.24	0.840	0.841	0.205	0.028	0.423	0.539	—

SD = standard deviation; α = Cronbach’s alpha; ω = McDonald’s omega. Correlations are Pearson’s coefficients. No validated cutoff points are available for these instruments, so scores are interpreted relative to the possible range.

Before estimating the structural relationships, the measurement properties of the five instruments were examined through confirmatory factor analysis. Table 1 presents the standardized factor loadings, composite reliability, average variance extracted and evidence of discriminant validity. Item-level loadings are reported in Supplementary Table S3.

The five-factor measurement model provided acceptable fit to the data (scaled χ^2^(541) = 1007.87, *p* < 0.001; CFI = 0.914; TLI = 0.906; RMSEA = 0.061, 90% CI [0.055, 0.067]; SRMR = 0.075). Standardized loadings ranged from 0.41 to 0.92 and were all statistically significant (*p* < 0.001), with two items of the work satisfaction scale loading below 0.50. These items (WS3: λ = 0.425; WS5: λ = 0.410) were retained to preserve the item composition of the validated Turkish version of the scale. The measurement model included nine residual covariances between items within the same scale, specified based on modification indices and content overlap between the items; all were statistically significant and are reported in Supplementary Table S4. Without these parameters, model fit was lower (CFI = 0.890; TLI = 0.881; RMSEA = 0.069; SRMR = 0.077). Composite reliability exceeded 0.70 for all five constructs. Average variance extracted exceeded 0.50 for workload, quiet quitting, and change fatigue, whereas the values for presenteeism and work satisfaction fell below this threshold; following Fornell and Larcker (54), convergent validity may still be regarded as adequate under these conditions because composite reliability exceeded 0.60. Discriminant validity was supported by both criteria: the square root of the average variance extracted exceeded all corresponding inter-factor correlations, and all heterotrait-monotrait ratios were below the 0.85 threshold, with the highest being 0.695 for workload and quiet quitting (Table 1).

Because all measures were self-reported and collected on a single occasion, common method variance was assessed before proceeding to structural analysis. The first unrotated component accounted for 33.67% of the total variance, below the 50% criterion, and five components had eigenvalues greater than 1. A confirmatory factor analysis specifying a single common factor for all 35 items fitted the data poorly (scaled χ^2^(560) = 3204.48, *p* < 0.001; CFI = 0.509; TLI = 0.478; RMSEA = 0.144; SRMR = 0.158) and was clearly inferior to the five-factor model. Common method variance is therefore unlikely to account for the observed associations.

Table 4 presents the direct, indirect and total effects estimated in the final path model. The model showed mixed fit: scaled χ^2^(3) = 8.75, *p* = 0.033; CFI = 0.956; TLI = 0.852; RMSEA = 0.076, 90% CI [0.019, 0.138]; SRMR = 0.043. CFI, RMSEA, and SRMR met the prespecified guidelines, whereas TLI was below the 0.90 criterion. The model explained 55.6% of the variance in quiet quitting. Change fatigue was positively associated with presenteeism (B = 0.703, 95% CI [0.433, 0.994]; β = 0.539) and with workload (B = 0.464, 95% CI [0.076, 0.831]; β = 0.185), whereas its direct association with quiet quitting was not statistically significant (B = 0.287, 95% CI [−0.233, 0.744]; β = 0.145). Workload was positively associated with quiet quitting (B = 0.465, 95% CI [0.385, 0.539]; β = 0.589) and negatively associated with work satisfaction (B = −0.133, 95% CI [−0.188, −0.077]; β = −0.242), and work satisfaction was in turn negatively associated with quiet quitting (B = −0.139, 95% CI [−0.235, −0.063]; β = −0.097). The direct association between presenteeism and quiet quitting was positive but marginal, as the bootstrap confidence interval marginally included zero (B = 0.353, 95% CI [−0.008, 0.680]; β = 0.233).

**Table 4:** Direct, indirect, and total effects in the final path model (*N* = 329).

Path/effect	B	SE	95% CI (bootstrap)	β
Presenteeism (R^2^ = 0.290)				
CF → PRE	0.703	0.139	[0.433, 0.994]	0.539
Workload (R^2^ = 0.034)				
CF → WL	0.464	0.187	[0.076, 0.831]	0.185
Work satisfaction (R^2^ = 0.058)				
WL → WS	−0.133	0.028	[−0.188, −0.077]	−0.242
Quiet quitting (R^2^ = 0.556)				
CF → QQ (direct)	0.287	0.237	[−0.233, 0.744]	0.145
PRE → QQ (direct)	0.353	0.175	[−0.008, 0.680]	0.233
WL → QQ (direct)	0.465	0.041	[0.385, 0.539]	0.589
WS → QQ (direct)	−0.139	0.043	[−0.235, −0.063]	−0.097
Indirect and total effects				
CF → PRE → QQ	0.248	0.126	[0.017, 0.556]	0.125
CF → WL → QQ	0.216	0.086	[0.043, 0.398]	0.109
CF → WL → WS → QQ	0.009	0.005	[0.002, 0.023]	0.004
CF total indirect	0.472	0.160	[0.188, 0.858]	0.239
CF total effect	0.759	0.212	[0.295, 1.148]	0.383
WL → WS → QQ	0.018	0.007	[0.008, 0.036]	0.023
WL total effect	0.484	0.039	[0.405, 0.556]	0.612
PRE total effect	0.353	0.175	[−0.008, 0.680]	0.233
WS total effect	−0.139	0.043	[−0.235, −0.063]	−0.097

Estimates were obtained from the simultaneous path model shown in Figure 1, estimated in lavaan with the robust maximum likelihood estimator (MLR). Confidence intervals are bias-corrected bootstrap intervals based on 5,000 resamples. B = unstandardized coefficient; SE = standard error; β = fully standardized coefficient; R^2^ = proportion of variance explained. Model fit: scaled χ^2^(3) = 8.75, *p* = 0.033; CFI = 0.956; TLI = 0.852; RMSEA = 0.076, 90% CI [0.019, 0.138]; SRMR = 0.043. Confidence intervals refer to the unstandardized coefficients.

Regarding the indirect pathways, change fatigue showed significant indirect associations with quiet quitting through presenteeism (B = 0.248, 95% CI [0.017, 0.556]; β = 0.125), through workload (B = 0.216, 95% CI [0.043, 0.398]; β = 0.109) and through the sequential pathway of workload and work satisfaction (B = 0.009, 95% CI [0.002, 0.023]; β = 0.004). Its total indirect effect (B = 0.472, 95% CI [0.188, 0.858]; β = 0.239) and total effect (B = 0.759, 95% CI [0.295, 1.148]; β = 0.383) were both significant. Workload also showed a small but significant indirect association with quiet quitting through work satisfaction (B = 0.018, 95% CI [0.008, 0.036]; β = 0.023), and its total effect was the largest in the model (B = 0.484, 95% CI [0.405, 0.556]; β = 0.612) (Table 4).

Because quiet quitting scores were concentrated near the upper end of the scale, three sensitivity analyses were conducted to assess the robustness of the estimated paths. Excluding the 15 multivariate outliers, adjusting for profession, and adjusting for sex, age, department, weekly working hours, weekly on-call duties, employment contract type, and professional experience left the associations of workload with quiet quitting (β = 0.748, 0.585, and 0.585, respectively), of workload with work satisfaction, and of work satisfaction with quiet quitting essentially unchanged, and none of the sociodemographic or occupational covariates was significantly associated with quiet quitting. The association between presenteeism and quiet quitting was the only path that was not robust: it decreased from β = 0.233 in the primary model to β = 0.076 (*p* = 0.201; 95% CI [−0.041, 0.200]) after excluding multivariate outliers. Inspection of these 15 cases showed that they were the participants reporting the lowest quiet quitting scores (mean = 2.71 versus 4.50 in the remaining sample), so excluding them substantially restricted the range of the outcome variable. The estimation of this particular path therefore depends on a small subgroup of participants at the lower end of a distribution that is otherwise close to ceiling, and this association was not robust to the exclusion of these cases. Detailed results of the sensitivity analyses are provided in Supplementary Table S1.

A multigroup analysis by professional group was also conducted. Constraining the structural paths to be equal across physicians, nurses and other healthcare professionals resulted in a substantial deterioration in fit relative to the configural model (CFI decreased from 0.949 to 0.718, and RMSEA increased from 0.105 to 0.154; scaled Δχ^2^(14) = 55.76, *p* < 0.001), and both AIC and BIC favored the unconstrained model. Path-specific equality tests indicated that this non-invariance was confined to the pathways involving change fatigue and presenteeism. The association between workload and quiet quitting did not differ statistically across groups (scaled Δχ^2^(2) = 0.84, *p* = 0.657) and was significant in all three (β = 0.598 for physicians, 0.711 for nurses and 0.346 for other professionals). The paths from change fatigue to workload (scaled Δχ^2^(2) = 1.04, *p* = 0.594) and from workload to work satisfaction (scaled Δχ^2^(2) = 0.44, *p* = 0.805) were likewise equivalent across groups, and the equality test for the path from work satisfaction to quiet quitting was marginal (scaled Δχ^2^(2) = 5.24, *p* = 0.073). In contrast, the associations of change fatigue with presenteeism (scaled Δχ^2^(2) = 17.84, *p* < 0.001) and with quiet quitting (scaled Δχ^2^(2) = 20.80, *p* < 0.001), together with the association between presenteeism and quiet quitting (scaled Δχ^2^(2) = 8.45, *p* = 0.015), differed across professional groups. Presenteeism was significantly associated with quiet quitting among nurses and other professionals but not among physicians, and change fatigue showed a significant direct association with quiet quitting among physicians and other professionals but not among nurses. Given the small size of the third group, these group-specific results should be regarded as exploratory. Full results are reported in Supplementary Table S2. Measurement invariance was not formally tested in the present study.

Regarding the study hypotheses, H1a, H1b, H2, H4, and H5 were supported. H1c was not supported, as the direct association between change fatigue and quiet quitting was not statistically significant. H3 received only marginal support, as the bootstrap confidence interval for the path from presenteeism to quiet quitting spanned zero, and this association lost statistical significance once multivariate outliers were excluded. Regarding the indirect pathways, H6a, H6b, H6c, and H7 were supported based on the bootstrap confidence intervals, although the effects specified in H6c and H7 were very small in magnitude and, as indicated by the Monte Carlo power analysis, were estimated with limited statistical power.

The final path model is presented in Figure 1, which displays the standardized coefficients reported in Table 4 together with the proportion of variance explained in each endogenous variable (Figure 1).

**Figure 1:**
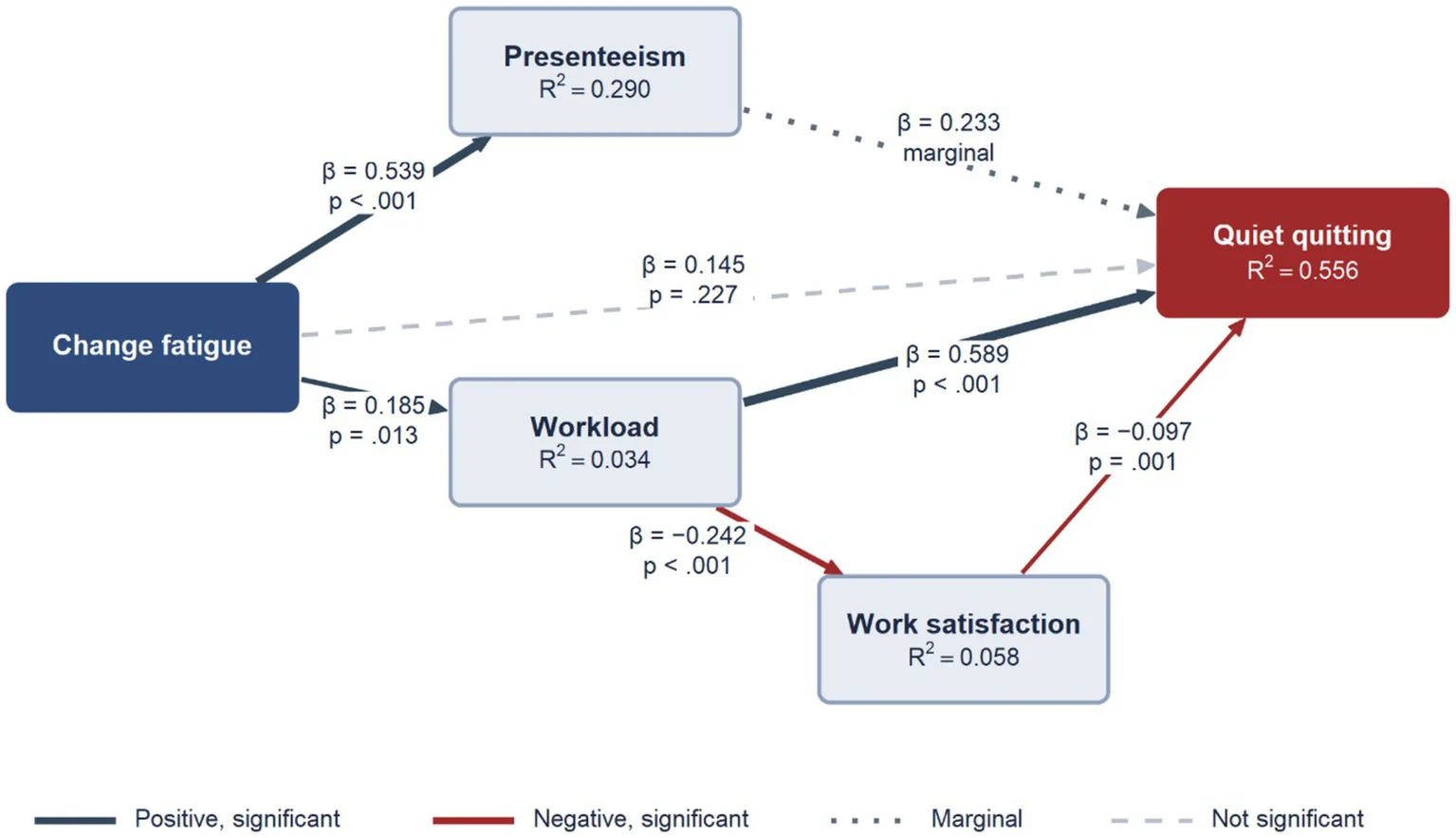
Structural equation model. Values displayed on the arrows are fully standardized path coefficients. R^2^ values represent the proportion of variance explained in each endogenous variable. Solid lines indicate statistically significant associations; the dotted line indicates the association between presenteeism and quiet quitting, for which the bootstrap confidence interval marginally included zero; and the dashed line indicates a non-significant association. Line thickness is proportional to the magnitude of the coefficient.

## 4 Discussion

This study examined the direct and indirect associations of workload, work satisfaction, change fatigue, and presenteeism with quiet quitting among 329 healthcare professionals employed at a public hospital in Denizli, Türkiye.

Workload showed the strongest association with quiet quitting in this sample, whereas the associations of presenteeism and change fatigue with quiet quitting were weaker and, for presenteeism, not robust across specifications. Considered against the study hypotheses, the associations of change fatigue with presenteeism and workload (H1a, H1b), of workload with quiet quitting (H2) and with work satisfaction (H4), and of work satisfaction with quiet quitting (H5) were all supported, as were the four indirect pathways (H6a–H6c, H7). The direct association between change fatigue and quiet quitting (H1c) was not supported, and the association between presenteeism and quiet quitting (H3) received only marginal support.

Change fatigue was strongly associated with presenteeism and, more weakly, with workload, and showed significant indirect associations with quiet quitting. With these indirect pathways included in the model, its direct association with quiet quitting was not statistically significant. This pattern is consistent with the Job Demands–Resources framework and suggests that workload and presenteeism may help explain the association between change fatigue and quiet quitting. However, the cross-sectional design does not establish the temporal order or causal mechanisms underlying these associations.

SEM analysis indicated that change fatigue was associated with quiet quitting indirectly through workload and presenteeism rather than directly. This aligns with prior studies showing that repeated organizational changes induce change fatigue, heighten perceived workload and uncertainty, and trigger resistance and withdrawal behaviors (19, 55). High workload has been identified as a key determinant of presenteeism among healthcare workers, with increased demands and stress contributing to burnout and reduced productivity despite physical presence at work (31, 56–58). Presenteeism has also been linked to emotional exhaustion, diminished organizational commitment, and heightened withdrawal tendencies, supporting its role as a psychological antecedent of quiet quitting (59–61). Recent work developed in the Turkish healthcare context has extended the concept of presenteeism beyond attending work while ill, defining it more broadly as reduced engagement and off-task behavior during working hours, and has shown it to be positively associated with burnout and negatively with happiness at work (62). In the present study, however, the direct association between presenteeism and quiet quitting was positive but marginal, and it was not robust after excluding participants with extreme response patterns. This finding therefore requires replication before presenteeism can be regarded as a firmly established correlate of quiet quitting in this population. Work satisfaction showed a negative association with quiet quitting, consistent with the view that higher satisfaction reinforces employee commitment. This finding is consistent with literature highlighting the role of satisfaction and organizational support in preventing quiet quitting, especially in high-stress healthcare environments (63–65). Overall, the study contributes to the literature by indicating that change fatigue is indirectly associated with quiet quitting through workload and presenteeism, emphasizing the need to examine these relationships holistically in healthcare contexts. The mean quiet quitting score in this sample (4.41 on a 1–5 scale, SD = 0.64) was close to the upper end of the scale, with the distribution concentrated in that region. As no validated cutoff points exist for this scale to classify scores as low, moderate, or high, we refrain from categorical labeling and instead interpret this finding descriptively, relative to the scale range and in comparison with other variables in the model. The elevated mean quiet quitting score, together with workload showing the strongest total association with quiet quitting in the structural model (β = 0.612), suggests that workplace demands may contribute to understanding the elevated scores observed in this sample.

The findings are also consistent with an interrelated set of processes linking workload, presenteeism, and work satisfaction, although no interaction effects were tested in the present model. Excessive workload has been associated with the depletion of physical and mental resources and, in turn, with burnout and presenteeism, in which employees are physically present but psychologically withdrawn (61, 66–70). These conditions have been accompanied by decreased job performance, loss of motivation, and reduced engagement. Presenteeism has likewise been associated with lower organizational productivity and a more negative work climate (31, 71, 72). Work satisfaction was negatively associated with quiet quitting, which is consistent with literature describing satisfaction as a protective resource (70, 73–75). Nevertheless, the magnitude of this association was small (β = −0.097) relative to workload, so the protective role of work satisfaction should not be overstated. In this sample, higher work satisfaction was associated with lower quiet quitting scores, although this association was modest. Whether work satisfaction buffers the association of workload with quiet quitting or influences pathways involving presenteeism requires further investigation.

The multigroup analysis indicated that these relationships did not operate uniformly across professional groups. The association between workload and quiet quitting was statistically equivalent across physicians, nurses, and other healthcare professionals and was significant in all three, suggesting that workload is a common correlate of quiet quitting irrespective of professional role. The pathways involving change fatigue and presenteeism, by contrast, differed across groups: change fatigue was strongly associated with presenteeism among physicians and nurses but not among the remaining professionals, and presenteeism was associated with quiet quitting among nurses and other professionals but not among physicians. These groups differ in working conditions, proximity to continuous patient care, and the degree of autonomy they exercise over their schedules, and these differences may plausibly shape how organizational change is experienced. Such differences may also arise when measurement properties are not fully invariant across groups, in which case group-specific coefficients partly capture measurement artifacts rather than structural differences (76), and measurement invariance was not formally tested here. Consequently, organizational policy interventions should not apply a one-size-fits-all approach; while workload reduction is universally beneficial, strategies addressing change fatigue and presenteeism must be customized to the distinct workplace dynamics of physicians and nurses.

These findings also bear on how quiet quitting is interpreted. The pattern observed here, in which quiet quitting was most strongly associated with workload and with the cumulative strain of organizational change, is more consistent with a response to working conditions than with an individual failing. Reduced discretionary effort may reflect an attempt to protect finite personal resources when demands exceed what can be sustained, and interventions directed at individual employees are unlikely to address the organizational conditions associated with quiet quitting in this study.

These results have practical implications for sustainable human resource management in healthcare. In this sample, workload was the variable most strongly associated with quiet quitting and was also negatively associated with work satisfaction. Given the central position of workload in the model, potential strategies could include regularly assessing staffing requirements, balancing task distribution, and monitoring overtime. Regarding presenteeism, occupational health measures, psychological support, adequate rest opportunities, and policies allowing staff to take leave when unfit to work may be relevant. During periods of organizational change, involving staff, communicating in advance, explaining the reasons for change, and considering the cumulative burden of multiple simultaneous changes may be helpful. Work satisfaction may be supported through recognition, managerial support, autonomy, organizational justice, and opportunities for professional development. These are presented as potential strategies only, since the effectiveness of such interventions was not evaluated in this study. However, this study has several limitations that warrant acknowledgment. First, its cross-sectional design precludes causal inferences regarding the relationships among the study variables. The direction of the observed relationships cannot be established, and reverse or reciprocal associations are equally consistent with the data; for example, professionals who have already withdrawn psychologically may perceive their workload more negatively and report lower work satisfaction. Second, because the study was conducted in a single public hospital in Türkiye, the findings should be interpreted with caution; they may not generalize to healthcare professionals in different hospitals, healthcare systems, or cultural contexts. Differences in organizational structures, staffing patterns, and institutional policies may influence these relationships. In addition, detailed institutional information on the nature, timing, duration, and scope of organizational changes was not collected. Therefore, the change fatigue findings reflect participants’ cumulative perceptions of organizational change rather than their responses to a specific change initiative. Third, the near-ceiling distribution of quiet quitting scores may have attenuated the detectable strength of some associations with other study variables, potentially underestimating their true magnitude. Fourth, because participation was voluntary, self-selection bias cannot be excluded, as professionals experiencing more pronounced disengagement may have been more inclined to participate. Moreover, given that data were collected over a specific 3-month period (July–September 2025), the generalizability of this particularly elevated mean to other seasonal or institutional contexts should be interpreted with caution. Fifth, the data departed from multivariate normality, which was addressed through robust estimation and bootstrapping but which may still affect the precision of some estimates. Sixth, the Monte Carlo power analysis indicated that although the study was well powered for the principal indirect associations, power was limited for the two smallest indirect associations involving work satisfaction; the absence of larger effects along these pathways should therefore not be interpreted as evidence that no such associations exist. Seventh, the average variance extracted for presenteeism and work satisfaction fell below the conventional threshold of 0.50, indicating that these two constructs were measured with greater error than the remaining three. In addition, two work satisfaction items had standardized factor loadings below 0.50, which warrants caution when interpreting findings involving this construct. Eighth, the structural relationships were estimated using composite scores rather than latent variables, which does not correct for measurement error, although this specification was chosen because the sample size did not support a fully latent model. Finally, the multigroup analysis indicated that the structural relationships differed across professional groups, so the pooled estimates should be understood as an average across professions rather than as a model that applies uniformly to physicians, nurses, and other healthcare professionals. Measurement invariance across professional groups was not formally tested, so the group-specific estimates should be interpreted with caution (76). Future multicenter, longitudinal studies in more diverse healthcare settings are recommended to improve the external validity of the findings. Despite these limitations, the study examines several organizational and psychosocial correlates of quiet quitting within a single model and identifies workload as the variable most consistently associated with it across specifications and professional groups.

## 5 Conclusion

In this cross-sectional study conducted in a single public hospital, workload was positively associated with quiet quitting and was the only association that remained robust across all model specifications and professional groups. Work satisfaction showed a small negative association with quiet quitting. The direct association between presenteeism and quiet quitting was not fully supported, as it was marginal and failed to remain significant after excluding multivariate outliers. Change fatigue was not directly associated with quiet quitting but was indirectly associated with it through workload and presenteeism. Furthermore, multigroup analysis indicated that the pathways involving change fatigue and presenteeism differed between physicians and nurses. Because the design is cross-sectional and confined to one institution, it cannot establish the temporal or causal direction of these relationships. Longitudinal and multicenter studies are needed to test the proposed model and to evaluate whether targeted organizational interventions reduce quiet quitting.

### 5.1 Limitations

This study has several limitations that should be acknowledged. First, its cross-sectional design precludes causal inferences regarding the relationships among the study variables. Second, because the study was conducted in a single public hospital in Türkiye, interpret the findings with caution; they may not generalize to healthcare professionals in different hospitals, healthcare systems, or cultural contexts. Differences in organizational structures, staffing patterns, and institutional policies may influence these relationships. In addition, detailed institutional information on the nature, timing, duration, and scope of organizational changes was not collected. Therefore, the change fatigue findings reflect participants’ cumulative perceptions of organizational change rather than their responses to a specific change initiative. Third, the near-ceiling distribution of quiet quitting scores may have attenuated the detectable strength of some associations with other study variables, potentially underestimating their true magnitude. Fourth, as participation was voluntary, self-selection bias cannot be excluded, as professionals experiencing more pronounced disengagement may have been more inclined to participate. Fifth, although supplementary models adjusted for profession and other sociodemographic and occupational characteristics, residual confounding cannot be excluded. Multigroup analyses were also conducted; however, the small size of the other healthcare professionals’ group and the absence of formal measurement invariance testing warrant cautious interpretation of group-specific findings. Finally, given that data were collected over a specific 3-month period (July–September 2025), the generalizability of the findings to other seasonal or institutional contexts should be interpreted with caution. Future studies with larger and more balanced professional subgroups, preferably using multicenter and longitudinal designs, should assess measurement invariance and further examine the robustness of the adjusted and group-specific associations.

Despite these limitations, the study provides a comprehensive model explaining quiet quitting among healthcare professionals and offers practical implications for organizational interventions to reduce workload, prevent presenteeism, and improve work satisfaction.

## Data Availability

The raw data supporting the conclusions of this article will be made available by the authors, without undue reservation.

## Supplementary material

TABLE S1 Sensitivity analyses: standardized coefficients from the primary path model and from three alternative specifications.

TABLE S2 Multigroup path analysis by professional group.

TABLE S3 Item-level standardized factor loadings for the five-factor measurement model.

TABLE S4 Residual covariances retained in the five-factor measurement model.
