# Supplementary material for: Associations of workload, work satisfaction, change fatigue, and presenteeism with quiet quitting among healthcare professionals: a structural equation modeling study
Source: Front Public Health. 2026 Oct 21;14:1936291. doi: 10.3389/fpubh.2026.1936291 (PMC13731387; doi:10.3389/fpubh.2026.1936291)
Supplement: Supplementary file 1 [file Supplementary_file_1.pdf]

Supplementary Material for

Associations of Workload, Work Satisfaction, Change Fatigue, and Presenteeism with Quiet Quitting Among Healthcare Professionals: A Structural Equation Modeling Study

Birol Yetim, Seval Selvi Sarıgül, İsmail Biçer and Yusuf Çelik*

*: Corresponding author, yusuf.celik@acibadem.edu.tr

Table S1. Sensitivity analyses: standardized coefficients from the primary path model and from three alternative specifications

Path or effect	Primary model	Outliers excluded	Profession adjusted	Fully adjusted
**Direct paths**				
CF → PRE	0.539 (<.001)	0.620 (<.001)	0.532 (<.001)	0.532 (<.001)
CF → WL	0.185 (.011)	0.179 (.008)	0.179 (.013)	0.179 (.013)
WL → WS	−0.242 (<.001)	−0.229 (<.001)	−0.241 (<.001)	−0.241 (<.001)
CF → QQ	0.145 (.222)	0.163 (.048)	0.143 (.221)	0.138 (.238)
PRE → QQ	0.233 (.039)	0.076 (.201)	0.232 (.042)	0.217 (.050)
WL → QQ	0.589 (<.001)	0.748 (<.001)	0.585 (<.001)	0.585 (<.001)
WS → QQ	−0.097 (.001)	−0.093 (.001)	−0.096 (.001)	−0.103 (<.001)
**Indirect and total effects**				
CF → PRE → QQ	0.125 (.039)	0.047 (.183)	0.123 (.041)	0.115 (.048)
CF → WL → QQ	0.109 (.011)	0.134 (.006)	0.105 (.012)	0.105 (.012)
CF → WL → WS → QQ	0.004 (.054)	0.004 (.040)	0.004 (.058)	0.004 (.044)
CF total indirect	0.239 (.002)	0.184 (.005)	0.232 (.002)	0.225 (.002)
CF total effect	0.383 (<.001)	0.347 (<.001)	0.375 (<.001)	0.362 (<.001)
WL → WS → QQ	0.023 (.005)	0.021 (.006)	0.023 (.006)	0.025 (.003)
WL total effect	0.612 (<.001)	0.769 (<.001)	0.608 (<.001)	0.610 (<.001)
**Model information**				
n	329	314	329	329
Free parameters	11	11	19	28
Observations per parameter	29.9	28.5	17.3	11.8
Scaled χ^2 (df)	8.75 (3)	2.09 (3)	8.08 (3)	57.07 (30)
CFI	0.956	1.000	0.972	0.906
TLI	0.852	1.009	0.835	0.831
RMSEA	0.076	0.000	0.072	0.052
SRMR	0.043	0.023	0.030	0.042

Values are fully standardized coefficients with p values in parentheses. All models were estimated in lavaan with the robust maximum likelihood estimator (MLR). The outlier-excluded model omits the 15 cases identified as multivariate outliers by Mahalanobis distance ($p < .001$, df = 5). The profession-adjusted model includes two profession dummy variables as predictors of all four endogenous variables. The fully adjusted model additionally includes sex, age, department, weekly working hours, weekly on-call duties, employment contract type and professional experience as predictors of quiet quitting; none of these covariates was significantly associated with quiet quitting. Because the first three models have only three degrees of freedom, the TLI is estimated with limited precision at this level of parsimony and should be interpreted alongside the remaining indices. CF = change fatigue; WL = workload; PRE = presenteeism; WS = work satisfaction; QQ = quiet quitting.

Table S2. Multigroup path analysis by professional group

Panel A. Standardized coefficients and explained variance by group

Path	Physicians (n = 146)	Nurses (n = 117)	Other (n = 66)
**Standardized coefficients**			
CF → PRE	0.541 (<.001)	0.753 (<.001)	0.171 (.467)
CF → WL	0.050 (.532)	0.214 (.043)	0.265 (.139)
WL → WS	−0.230 (.006)	−0.200 (.013)	−0.340 (<.001)
CF → QQ	0.246 (.011)	−0.191 (.256)	0.386 (.011)
PRE → QQ	0.256 (.097)	0.330 (.029)	0.444 (.021)
WL → QQ	0.598 (<.001)	0.711 (<.001)	0.346 (.026)
WS → QQ	−0.028 (.437)	−0.125 (.006)	−0.165 (.039)
**Explained variance (R^2)**			
Presenteeism	0.293	0.567	0.029
Workload	0.002	0.046	0.070
Work satisfaction	0.053	0.040	0.116
Quiet quitting	0.584	0.625	0.689

Panel B. Path-specific tests of equality across groups

Path constrained equal across groups	Scaled $\Delta\chi^2$	df	p
CF → PRE	17.84	2	<.001
CF → WL	1.04	2	.594
WL → WS	0.44	2	.805
CF → QQ	20.80	2	<.001
PRE → QQ	8.45	2	.015
WL → QQ	0.84	2	.657
WS → QQ	5.24	2	.073
All structural paths	55.76	14	<.001

Values in Panel A are fully standardized coefficients with p values in parentheses, obtained from the configural model in which all structural paths were freely estimated across groups. In Panel B, each row reports the scaled chi-square difference obtained when the corresponding path is released from a model in which all structural paths are constrained equal across the three groups; the final row reports the test of the fully constrained model against the configural model. Configural model: CFI = 0.949; RMSEA = 0.105; SRMR = 0.042; AIC = 6797.6; BIC = 6968.4. Fully constrained model: CFI = 0.718; RMSEA = 0.154; SRMR = 0.126; AIC = 6878.8; BIC = 6996.5. Given the size of the third group, the group-specific estimates should be regarded as exploratory. Measurement invariance across professional groups was not formally tested. CF = change fatigue; WL = workload; PRE = presenteeism; WS = work satisfaction; QQ = quiet quitting.

Table S3. Item-level standardized factor loadings for the five-factor measurement model

Factor	Item	λ	SE	z
	WL1	.791	.031	25.50
	WL2	.798	.030	26.64
	WL3	.750	.064	11.76
	WL4	.840	.022	37.70
	WL5	.823	.022	36.72
**Workload**	WL6	.836	.026	32.31
	WL7	.625	.080	7.86
	WL8	.731	.068	10.76
	WL9	.852	.022	38.20
	WL10	.824	.031	26.89
	WL11	.608	.084	7.24
	WS1	.798	.042	19.17
	WS2	.711	.042	17.04
**Work satisfaction**	WS3	.425	.082	5.17
	WS4	.621	.042	14.91
	WS5	.410	.086	4.75
	QQ1	.776	.044	17.66
	QQ2	.761	.049	15.66
	QQ3	.844	.040	21.14
**Quiet quitting**	QQ4	.802	.042	19.22
	QQ5	.860	.025	33.89
	QQ6	.846	.028	30.60
	QQ7	.804	.050	16.11
	CF1	.802	.068	11.71
	CF2	.808	.052	15.61
**Change fatigue**	CF3	.765	.064	11.90
	CF4	.915	.024	37.68
	CF5	.855	.036	23.44
	CF6	.779	.047	16.70
	PRE1	.551	.078	7.02
	PRE2	.690	.039	17.86
**Presenteeism**	PRE3	.701	.040	17.50
	PRE4	.700	.044	16.02
	PRE5	.757	.035	21.46
	PRE6	.696	.043	16.23

N = 329. Estimated in lavaan with the robust maximum likelihood estimator (MLR). All loadings are statistically significant at $p < .001$. λ = standardized factor loading; SE = standard error. The model includes nine residual covariances between items belonging to the same scale, which are reported in Table S4. Model fit with these parameters was scaled $\chi^2(541) = 1007.87$, CFI = 0.914, TLI = 0.906, RMSEA = 0.061, SRMR = 0.075; without them, scaled $\chi^2(550) = 1147.46$, CFI = 0.890, TLI = 0.881, RMSEA = 0.069, SRMR = 0.077.

Table S4. Residual covariances retained in the five-factor measurement model

Scale	Item pair	Estimate	SE	z
**Workload**	WL1 – WL2	.297	.098	3.02
	WL2 – WL4	.250	.085	2.94
	WL5 – WL9	.269	.081	3.31
	WL8 – WL9	.251	.078	3.23
	WL8 – WL11	−.292	.063	−4.66
**Work satisfaction**	WS2 – WS3	.154	.055	2.81
	WS3 – WS5	.489	.055	8.89
	WS4 – WS5	.159	.061	2.62
**Change fatigue**	CF1 – CF3	.241	.121	1.99

N = 329. Values are standardized residual covariances between items belonging to the same scale, estimated in lavaan with the robust maximum likelihood estimator (MLR). All parameters are statistically significant ($p \leq .046$). These parameters were specified on the basis of modification indices together with content overlap between the items concerned. An initial specification containing four additional residual covariances was not retained because those parameters were not statistically significant; the two specifications differed negligibly in fit (CFI = 0.919 versus 0.914; TLI = 0.910 versus 0.906; RMSEA = 0.060 versus 0.061).
